# Category Theory for Autonomous and Networked Dynamical Systems

**DOI:** 10.3390/e21030302

**Published:** 2019-03-20

**Authors:** Jean-Charles Delvenne

**Affiliations:** Institute of Information and Communication Technologies, Electronics and Applied Mathematics (ICTEAM) and Center for Operations Research and Econometrics (CORE), Université catholique de Louvain, 1348 Louvain-la-Neuve, Belgium; jean-charles.delvenne@uclouvain.be

**Keywords:** ergodic theory, topological dynamics, control theory

## Abstract

In this discussion paper we argue that category theory may play a useful role in formulating, and perhaps proving, results in ergodic theory, topogical dynamics and open systems theory (control theory). As examples, we show how to characterize Kolmogorov–Sinai, Shannon entropy and topological entropy as the unique functors to the nonnegative reals satisfying some natural conditions. We also provide a purely categorical proof of the existence of the maximal equicontinuous factor in topological dynamics. We then show how to define open systems (that can interact with their environment), interconnect them, and define control problems for them in a unified way.

## 1. Introduction

The theory of autonomous dynamical systems has developed in different flavours according to which structure is assumed on the state space—with topological dynamics (studying continuous maps on topological spaces) and ergodic theory (studying probability measures invariant under the map) as two prominent examples. These theories have followed parallel tracks and built multiple bridges. For example, both have grown successful tools from the interaction with information theory soon after its emergence, around the key invariants, namely the topological entropy and metric entropy, which are themselves linked by a variational theorem.

The situation is more complex and heterogeneous in the field of what we here call open dynamical systems, or controlled dynamical systems. These are systems that can be controlled by an external time-varying input, and in return can influence the environment, or be observed, only through an output signal that typically represents partial information on the state. In particular the interaction with information theory has only been developed systematically since around 2000, and has given way to a flurry of concepts such as the topological feedback entropy [1], the invariance entropy [2], the anytime capacity [3], etc. Most of them capture the minimum amount of information that must be extracted in the interaction with an open system in order to achieve a certain task, for example maintain the state in a neighbourhood or a small average distance to a state of interest.

As open dynamical systems can naturally be interconnected, not just in two (e.g., a given plant to be controlled and a controller, designed especially in the goal to drive or observe the plant) but in arbitrary networks of given plants and to-be-designed controllers, we can expect an extension of the key concepts to an even wider diversity, depending on the assumptions on the goal or shape of the networking.

While this diversity is the sign of a rich topic and a broad range of potential applications, it is also interesting if one can bring an unifying view on the interaction between open systems theory and information theory, but also on the relation of those concepts with the corresponding concepts in autonomous systems theory.

While we do not claim to bring answers in this short discussion paper, we do propose some unifying ideas and formulate in this framework some elementary observations that may indicate the existence of deeper unifying theorems ahead. In this, we hope that this discussion paves the way for a new line of research, not only in the theory of information-limited open dynamical systems, but also possibly in the older fields of ergodic theory and topological dynamics.

A key tool that we would like to discuss here is category theory, which has proved a spectacular unifying power in topology, algebraic geometry, logic, combinatorics, etc.

Another key idea is the one of behaviour, which has been introduced by Willems et al. in the deterministic setting as the set of all possible joint trajectories of the input and output variables that are compatible with the internal constraints of the open dynamical system, which allows to formulate a large part of deterministic (non-random) open systems theory in an elegant way. There have been few attempts to extend the theory of behaviours in a stochastic setting, however, and we propose a solution to fill this gap.

The paper is organized as follows. Firstly, we introduce a vanilla version of categories for topological dynamics and ergodic theory, with a purely categorical derivation of Shannon entropy and Kolmogorov–Sinai entropy as the unique concept satisfying some natural properties—an alternative to the usual axiomatic characterizations of Shannon entropy. Secondly, we observe that the maximal equicontinuous factor in topological dynamics can also be obtained very simply in a categorical setting—as a consequence of the celebrated adjoint functor theorem. Thirdly, we introduce the category of stochastic behaviours, which we claim allows a natural formulation of classical results such as the variational theorem, and serves as a guide for a formulation of stochastic behaviours. The categorical setting offers a clean way to define the interconnection of stochastic behaviours, and offers a natural notion of entropy for networks of systems, which we briefly explore on a simple example of a linear system.

The main text focusses on the discussion of the definition and the observations, while the technical proofs are concentrated into Appendix A and Appendix B.

## 2. A Categorical Characterization of Shannon Entropy and Kolmogorov–Sinai Entropy

A category is a class of objects and arrows linking the objects. Any arrow f:A→B from an object *A* to an object *B* can be composed with any arrow g:B→C in order to form an arrow g∘f:A→C: this is the composition. The composition is supposed to be associative and possess a neutral element: the identity arrow on each object. For example, groups (as objects) and group homomorphisms (as arrows) form a category of interest, as well as the category of sets and applications, the category of real vector spaces and linear applications, etc.

The objects of a category may form a set, as defined as in axiomatic set theory, or a proper class. For example the objects of the category of sets, or the category of groups, do not form a set but a proper class, as we know there is no such thing as a ‘set of all sets’, ‘set of all groups’, etc. There are also interesting ‘small’ categories, i.e., whose objects form a set rather than a proper class. For instance any set endowed with a preorder can be turned into a category, whose objects are the elements, and there exists a (single) arrow from *x* to *y* if x≤y. In this case the properties of composition represents the transitivity and reflexivity of the preorder. Note that in any category, small or large, the arrows between any two given objects are assumed to form a set (rather than a proper class). See [4] for a more extended discussion on these foundational issues.

Many concepts familiar in a set-theoretic view can now be transposed naturally in the categorical setting. For instance, two objects are called isomorphic if they are linked by a pair of arrows in opposite directions whose composition makes the identity on each object.

The main text of the article tries to keep an introductory level in terms of categorical aspects, at the expense of formal definitions or theorems. In contrast, the Appendix A and Appendix B does assume familiarity with the basics of category theory, and provides the technical proofs whenever needed.

Let us call **Prob** the category of probability spaces, i.e., sets endowed with a σ-algebra of events (or measurable sets) and a probability measure on these events. The arrows, or morphisms, are chosen to be the measure-preserving maps, i.e., the measurable maps such that the inverse image of an event is an event of the same probability.

Similarly we can define **Erg**, the category of endomorphisms of **Prob**. An endomorphism is defined as an arrow from an object to itself, thus a measure-preserving transformation of a probability space. An arrow in **Erg** is the usual notion of factor map of ergodic theory, i.e., a measure-preserving map between two probability spaces that commutes with the transformations of the two spaces.

Now one can immediately build functors from **Prob** to **Erg**.

A functor *F* between two categories is the association of each object *A* of the source category with an image object F(A) of the target category, so that each arrow f:A→B is sent to an image arrow F(f):F(A)→F(B). A functor is required to preserve composition between arrows and the identity arrow on each object.

An obvious functor is the identity arrow functor, which sends each probability space *A* to the trivial dynamical system (A,Id). A more interesting one is what we call the Bernoulli functor, sending *A* to the Bernoulli system $\left(A^{Z},\sigma \right)$, where σ is the Bernoulli shift on the bi-infinite sequences of elements of *A*, endowed with the product algebra of events.

Before going further, we like to notice here that those two categories have another interesting structure: they are monoidal. A monoidal category C is a category endowed with a notion of product, usually called the tensor product, as a functor ⊗:C×C→C. This product must satisfy some conditions, such as isomorphism between (A⊗B)⊗C and A⊗(B⊗C), and the existence of a neutral object *I*, in the sense that A⊗I is isomorphic to *A* and to I⊗A. Those conditions also include the naturalness of these isomorphisms; for example, any arrow $f:A\to A^{\prime }$ induces an arrow $f⊗1_{I}:A⊗I\to A^{\prime }⊗I$ (with $1_{I}$ being the identity arrow on *I*), and we want these arrows to form a commutative square with the isomorphisms linking *A* with A⊗I and $A^{\prime }$ with $A^{\prime }⊗I$. Another condition is for instance that for any objects *A*, *B*, the three isomorphisms defined between (A⊗I)⊗B, A⊗(I⊗B) and A⊗B form a commutative triangle.

Note that as often in category theory, the tensor product is essentially defined up to isomorphism: another tensor product creating isomorphic objects is considered as essentially the same tensor product. If we take the skeleton of a category, i.e., a subcategory where we pick a representative object for each isomorphy class of objects, so that no two objects of the skeleton are isomorphic, then we see that the objects of a skeleton form a monoid in the usual sense.

For example, the category of finite sets, which is monoidal for the direct product, generates (N,1,×) as a monoid. The same category is also monoidal for the disjoint sum, with (N,0,+) as the corresponding monoid.

The usual direct product of two probability measures (joining them as independent marginals of a probability distribution on the product measurable space) acts as a tensor product on **Prob**, as well as on **Erg**, where the transformation is the direct product of the transformations on the individual spaces.

We can now formulate Ornstein’s celebrated isomorphism theorem on Bernoulli shifts in this framework. According to Ornstein’s theorem, any two Bernoulli shifts with the same Kolmogorov–Sinai (KS) entropy are isomorphic. We consider **Ber**, the category of Bernoulli shifts and factor maps, which is the image of the Bernoulli functor and also a subcategory of **Erg**, thereby inheriting the monoidal structure. Ornstein’s isomorphism theorem therefore states that the monoid corresponding to the subcategory of Bernoulli shifts endowed with the tensor product is simply the nonnegative real numbers with the addition. Moreover, from Ornstein’s theory (and in fact Sinai’s factor theorem) there is an arrow between two Bernoulli shifts if and only if the source of the arrow has a larger entropy than the target.

We consider the nonnegative reals (extended with +∞) as a monoidal category with the usual ≥ relation to define the arrows and the addition as tensor product. We can thus build a functor from the Bernoulli shifts to the nonnegative real numbers, that sends every Bernoulli shift *B* to its KS entropy h(B), and sends every arrow (factor map) f:A→B to the unique arrow h(A)→h(B) encoding the fact that h(A)≥h(B). This KS-entropy functor also respects the tensor product of each category.

It is easy to see that there is only one such essentially tensor-product-preserving tensor to the category of nonnegative reals, up to a trivial scaling by a scalar multiplicative constant (and avoiding the trivial all-to-zero functor)—see Appendix A for a formal statement and proof. Therefore KS entropy can be defined in a purely categorical manner, as the only such functor, without any reference to information theory.

Composing the Bernoulli functor from **Prob** to **Erg** with the KS-entropy functor, we obtain the Shannon entropy functor, which again is defined in a purely categorical way, without any prior reference to a $\sum_{i}p_{i}\log p_{i}$-like formula. In that, it offers an alternative to the derivation of Shannon entropy as the unique quantity (up to a multiplicative constant) that satisfy a list of natural axioms [5,6]. It also complements the categorical interpretation of entropy by Baez et al. [7], which characterizes the loss of Shannon entropy along an arrow in **Prob** as a functor to the category of one object with all reals as arrows. Let us finally mention Gromov’s categorical approach to Shannon entropy in his discussion paper [8], based on a formalization of the asymptotic equipartition property (Shannon–McMillan–Breiman). Interestingly, the latter is also one of the ingredients of Ornstein’s theorem.

Note that Sinai’s factor theorem allows us to extend the KS-entropy functor to all **Erg**, as the smallest possible such extension. This is again a specific instance of a general construction allowing to extend a functor from a category to a wider domain, called the Kan extension. Again we observe we can therefore define KS entropy for any system in **Erg** as the unique functor satisfying some structural properties.

Rather than offering a new practical definition for entropies, the point of these results is rather to argue that classical results of ergodic theory have a simple and natural expression in categorical terms. A natural follow-up would be to look for a categorical proof of those results. Although this is a far-reaching programme, we show in the next section how category theory can help re-derive some non-trivial facts of dynamical systems.

## 3. Adjoint Functors for Dynamical Systems

A key concept of category theory is the search for adjoint functors, i.e., functors that travel in reverse directions between two categories, and are in some sense as close as possible to being ‘inverse’ functors.

For instance, the category **HausComp** of Hausdorff compact spaces and continuous maps is a subcategory of the category **Top** of topological spaces and continuous maps. Therefore we have the trivial inclusion functor F:HausComp→Top. On the other hand, Stone–Čech compactification offers a functor G:Top→HausComp.

This pair of functors share the following property. Given an object *A* in **Top**, there is an arrow $f_{A}:A\to FGA$ such that any arrow from *A* to an object FX (i.e., to a compact Hausdorff space) factors uniquely as $Fg\circ f_{A}$ for some (unique) arrow g:GA→X. In other words, the adjointness property in this case says that the Stone–Čech compactification is the ‘mother of all compactifications’ of *A*: any other continuous map from *A* to a Hausdorff compact space factors through $f_{A}:A\to FGA$. In category theory, this is formalized in saying that *G* is a left adjoint to *F*, and *F* is a right adjoint to *G*, denoted G⊣F.

Another example is the construction of free groups in a categorical manner. The ‘forgetful’ functor F:Grp→Set which maps a group to its set of elements, ‘forgetting’ the group structure, has a left adjoint G:Set→Grp which maps a set to the free group generated from that set.

In both cases, the adjoint functor to the trivial functor corresponds to a fundamental construction in the corresponding theory. Once again, we do not claim to cover exhaustively the theory of adjoint functors here, only to give an informal foretaste.

It is therefore natural, given a functor between two categories, to ask whether it has an adjoint, in the hope to discover some fundamental construction between categories. The so-called general adjoint functor theorem provides a convenient set of sufficient conditions for a functor to have an adjoint. Remarkably, both the free group functor and the Stone–Čech Compactification functor above are constructed from their corresponding trivial right adjoint functor from this theorem. One can see how different, non-trivial construct can be obtained from a single category-theory argument.

We illustrate the potential usefulness of the general adjoint functor theorem in dynamical systems theory with a single example. Topological dynamics is captured with a category **TopDyn** of continuous transformations of Hausdorff compact spaces as objects, and continuous maps commuting with the transformations as arrows.

An interesting subcategory in **TopDyn** is the category **EquiDyn** of equicontinuous systems, which are maps f:X→X such that *f*, $f^{2}$, $f^{3}$, etc. form an equicontinuous family for the unique uniformity compatible with the compact Hausdorff space *X*. This is equivalent to saying that for any neighbourhood *V* of the diagonal in the Cartesian square X×X, there is a neighbourhood *U* of the diagonal such that if (x,y)∈U then $(f^{n}\left(x\right),f^{n}\left(y\right))\in V$ for all n≥0. In intuitive words, if two points are close enough, then the trajectories generated from these points will remain close at all future times.

It so happens that the general adjoint functor theorem can be easily applied to the inclusion functor of **EquiDyn** into **TopDyn**, yielding a reverse functor mapping each topological dynamical system *A* to a specific equicontinuous system FGA, through which every morphism from *A* to an equicontinuous system factors uniquely. This FGA is none but the standard maximal equicontinuous factor of *A*. As far as we know this application of the general adjoint functor theorem was not noticed before. Moreover, in this proof we obtain more than the existence of a maximal equicontinuous factor: the functor also transforms homomorphisms between two topological dynamical systems into a homomorphism between their corresponding maximal equicontinuous factor.

The key properties that make the general adjoint functor theorem work in this case are the fact that arbitrary subsystems and products of equicontinuous systems are equicontinuous.

In the next section, we return to measure-theoretic systems and build a slightly richer category than **Erg**.

## 4. The Category of Stochastic Behaviours

The term ‘behaviour’ in the context of open dynamical systems (i.e., subject to mutual interaction with its environment) was coined by Jan Willems [9], and defined in the following way.

Consider an open deterministic dynamical system on the time line Z which shares with its environment some variables *z*, taking values at all times in a set *Z*, with its environment. This dynamical system is described in a behavioural way by a set of trajectories $B\subseteq Z^{Z}$. Any subset $Z^{Z}$ may be thought of as describing a valid dynamical system, even though we usually request more properties, such as being shift-invariant, or being closed under linear combinations (if *Z* is a vector space), etc.

This is to be contrasted with the mainstream input-state-output modelling, where some variables *u* (the ‘input’), *x* (the ‘state’) and *y* (the ‘output’) are defined, and an evolution relation of the type $x_{t+1}=f\left(x_{t},u_{t},t\right),y_{t}=g\left(x_{t}\right)$ is defined. This model can be turned into a behavioural description by taking *B* as the solution set, i.e., all trajectories ${\left(u_{t},y_{t}\right)}_{t\in Z}$ for which there exists a trajectory ${\left(x_{t}\right)}_{t\in Z}$ such as the dynamical equation is satisfied. In this case the variable set shared with the environment is z=(u,y).

A feedback interconnection is the connection of two systems whose variables shared with the environment live in the same space *Z*. In the behavioural setting, the system resulting from the interconnection of two systems $B_{1}$, $B_{2}$, both subsets of $Z^{Z}$, is simply the intersection $B_{1}\cap B_{2}$. In the input-state-output modelling, an interconnection is usually imposed by an explicit relation between the input of one system and the output of the other system.

One advantage of the behavioural approach is the conceptual simplicity that makes it easy to generalize, and that it does not require the distinction between input variables and output variables.

Little has been done to adapt the behavioural framework for open stochastic systems. We propose to remedy this gap in the following way.

A stochastic behaviour (X,f,M) is a measurable space *X*, endowed with a measurable transformation *f* and a convex (possibly empty) set of probability measures *M* that are all invariant for the transformation.

For example, the behaviour representing the Gaussian channel $y_{t}=u_{t}+\eta_{t}$, for the unit Gaussian white noise $\eta_{t}$, is given by the stochastic behaviour composed of ${\left(R^{2}\right)}^{Z}$, understood as the set of all possible sequences ${\left(u_{t},y_{t}\right)}_{t\in Z}$, endowed with the usual σ-algebra, the usual shift map, and all shift-invariant measures such that $y_{t}-u_{t}$ is distributed as a white Gaussian process.

Stochastic behaviours, as we have defined, form the objects of a category **StoBeh** of which we now define the arrows, or homomorphisms. An arrow between (X,f,M) and (Y,g,N) is given by a measurable map h:X→Y forming a commutative square with *f* and *g* (h∘g=f∘h), and mapping the measures of *M* to measures in *N*.

Of course **Erg** is a subcategory of **StoBeh**, as being the objects (X,f,M) where *M* is reduced to a single measure.

Beyond the ability to model open dynamical systems, **StoBeh** also has some nice categorical properties, including categorical products, and more generally all (small) limits.

Generally speaking, the categorical product of two objects *A* and *B* in a category is an object A×B with two arrows (the ‘projections’) from A×B to *A* and *B* such that any object *C* with any two arrows to *A* and to *B* factors through A×B: there is a unique arrow f:C→A×B such that all diagrams commute. In the category of sets, groups, real vector spaces, σ-algebras, and many others, this corresponds to the usual Cartesian product structure.

In the category of **Erg**, however, the direct product is not the categorical product. Indeed given two dynamical systems (X,f,μ) and (Y,g,ν), there may be many joinings (X×Y,f×g,λ) that project back to μ and ν, and are not the direct product measure μ×ν.

In **StoBeh**, the categorical product exists and is given by the set of all possible joinings. Specifically, given (X,f,M) and (Y,g,N), the product is given by (X×Y,f×g,L) and obvious projections maps, where *L* is the set of all measures that are invariant for f×g and project to a measure in *M* and a measure in *N*. It is easy to check that this is indeed the categorical product.

In fact, **StoBeh** also possesses infinite products, and more general constructions such as pullbacks, and more generally the constructions called categorical limits. The pullback of two arrows f:A→B and $f^{\prime }:A^{\prime }\to B$ is a pair of arrows g:C→A and $g^{\prime }:C\to A^{\prime }$ from some object *C*, creating a commutative square, and universal in the sense that any other pair of arrows with creating a commutative square with $f,f^{\prime }$ factors through *C* and $g,g^{\prime }$. A useful particular case is when $f,f^{\prime }$ are inclusion maps of $A,A^{\prime }$ into one same behaviour *B*: in this case the pullback is simply the intersection of *A* and $A^{\prime }$.

In the same way, **Prob** does not possess categorical limits but the category of convex sets of probability measures, which we name **Probs**, has products (as sets of joinings over the product space) and all limits.

The entropy functor defined above extends seamlessly to **StoBeh** as the supremum of KS entropy over all measures of the behaviour. It is now additive for the categorical product.

Note that there is a useful ‘full behaviour’ functor from **TopDyn** to **StoBeh**, taking a topological space and its continous transformation to the full behaviour of all Borel measures invariant for the transformation.

## 5. Interconnection of Stochastic Behaviours

We now show how to interconnected stochastic behaviours. Consider once again the behaviour $B_{1}$, solution of the equation $y_{t}=u_{t}+\eta_{t}$. Consider on the other hand the deterministic system described by $y_{t+1}^{\prime }=-y_{t}^{\prime }/2+u_{t}^{\prime }$. This system is described by the behaviour $B_{2}$ taking place on the set ${\left(R^{2}\right)}^{Z}$ of all trajectories ${\left(u_{t}^{\prime },y_{t}^{\prime }\right)}_{t\in Z}$, endowed with the shift map and all shift-invariant measures supported on the set of trajectories verifying the equation.

Now we want to interconnect the behaviours with the equations $y_{t}=u_{t}^{\prime },u_{t}=y_{t}^{\prime }$. How do we express this in the categorical language? We consider the full behaviour $B_{\mathrm{full}}$ of all shift-invariant probability measures on ${\left(R^{2}\right)}^{Z}$. Then we have the appropriate inclusion arrows $B_{1}\to B_{\mathrm{full}}$ and $B_{2}\to B_{\mathrm{full}}$. The pullback of those two arrows is simply the subobject $B_{\mathrm{inter}}$, intersection of $B_{1}$ and $B_{2}$ which are embedded into the common object $B_{\mathrm{full}}$.

The interconnected behaviour will be what we expect, namely the set of shift-invariant probability measures on ${\left(y_{t},y_{t}^{\prime }\right)}_{t\in Z}$ such that $y_{t+1}^{\prime }-y_{t}^{\prime }/2$ is white Gaussian and $y_{t+1}^{\prime }+y_{t}^{\prime }/2=y_{t}$.

Let us explore another type of interconnections. Take for example the absence of any interconnection, i.e., two behaviours *A* and $A^{\prime }$ on which we impose no shared variables. Then the ‘interconnection’ is simply the categorical product $A\times A^{\prime }$, which is the set of joinings compatible with the given marginals. Note the possible dependence between the processes, due for example to anterior preparation of states, even though there is no forced dynamical interaction.

Yet another example is the composition of two behaviours in series. Imagine a behaviour *B* on variables ${\left(u_{t},y_{t}\right)}_{t\in Z}$ and another behaviour $B^{\prime }$ on variables ${\left(u_{t}^{\prime },y_{t}^{\prime }\right)}_{t\in Z}$. We now interconnect these behaviours with $y_{t}=u_{t}^{\prime }$. Then we can draw the projection arrows $B\to B_{y}$, where $B_{y}$ is the behaviour of all possible marginal probability measures on ${\left(y_{t}\right)}_{t\in Z}$. Similarly, we consider the projection $B^{\prime }\to B_{u}^{\prime }$. The two behaviours can be both embedded in the full behaviour $B_{\mathrm{full}}$ of all shift-invariant measures on the one-dimensional trajectories $R^{Z}$, with arrows $B_{y}\to B_{\mathrm{full}}$ and $B_{u}^{\prime }\to B_{\mathrm{full}}$. Composing the arrows, one finds two arrows $B\to B_{\mathrm{full}}$ and $B^{\prime }\to B_{\mathrm{full}}$. Once again, the pullback of these two arrows—now not simply an intersection—provides the interconnected behaviour on the trajectories ${\left(u_{t},y_{t},y_{t}^{\prime }\right)}_{t\in Z}$.

In general we see that the interconnection of behaviours is given by a diagram with arrows representing the constraints of interconnections, and is solved by taking the limit of the diagram.

## 6. Control Objectives as Stochastic Behaviours

Interconnection through categorical limits can also be used to formulate control objectives and controller design problems. For instance, suppose that we wish to control a system with a direct, memory-less feedback $y_{t}=k\left(u_{t}\right)$, for some measurable function *k*. Then we create the behaviour *D* of all shift-invariant measures on trajectories $\left(u_{t},y_{t}\right)$, such that for each measure in *D*, there exists a *k* such that $y_{t}=k\left(u_{t}\right)\forall t\in Z$.

Taking now a ‘plant’ behaviour *B* on variables, we interconnect it with *D* (intersection of behaviours embedded in the full behaviour) to obtain all measures that are possible to reach for *B* while using a controller in *D*.

Suppose now that we define $B^{\prime }$, on the same variables as *B*, that models all possible measures that have a certain desirable property in certain context. For example we may take in $B^{\prime }$ all shift-invariant measures supported on a certain $K^{Z}$, for a certain compact set $K\subseteq R^{2}$. This behaviour models a situation of invariance control, where we want the trajectories at all times to remain in *K*. To avoid the emergence of unpractical solutions such as a measure concentrated on an unstable fixed point of the dynamics, we may also restrict the measures in $B^{\prime }$ to have a support of non-empty interior.

We now further interconnect (intersect, in this case) $B^{\prime }$ with *B* and *D*, to obtain the part of the behaviour of *B* that satisfies the control objective expressed with $B^{\prime }$ with the class of controllers modelled in *D*. The elegance of the behavioural framework is that it places all the ingredients of the control problem—plant, controller, objective—as objects of same type.

## 7. Entropy of Stochastic Behaviours

We now ask what notion of ‘entropy’ is appropriate for **StoBeh**. An obvious choice for a behaviour consisting of a convex family of measures *M* is the supremum of the KS entropy over *M*.

For example, this choice allows to define topological entropy in **TopDyn** as the composition of the full behaviour functor from **TopDyn** to **StoBeh** with the entropy functor from **StoBeh** to [0,+∞], in virtue of the variational theorem.

The entropy functor is also monoidal for the categorical product on **StoBeh** and the addition on [0,+∞].

Let us now discuss the potential meaning of this entropy for control problems.

Consider that we want to control a plant *B* to be into a certain class of measures contained in $B^{\prime }$, which we take as above as all shift-invariant measures staying in *K* at all times, with a controller in a certain class *C*, for instance deterministic controllers with a finite range for the input they feed to the plant (‘quantized controllers’).

The interconnection of *B*, $B^{\prime }$ and *C*, revealing all solutions of the control problem, is again a behaviour, of which we can consider the entropy, i.e., the supremum of KS entropy rates. We may wonder if this entropy has a particular meaning for the control problem, for example akin to the invariance entropy [2], or other measures counting the information rate through the controller needed to maintain invariance.

## 8. Conclusions

**Figure 1:**
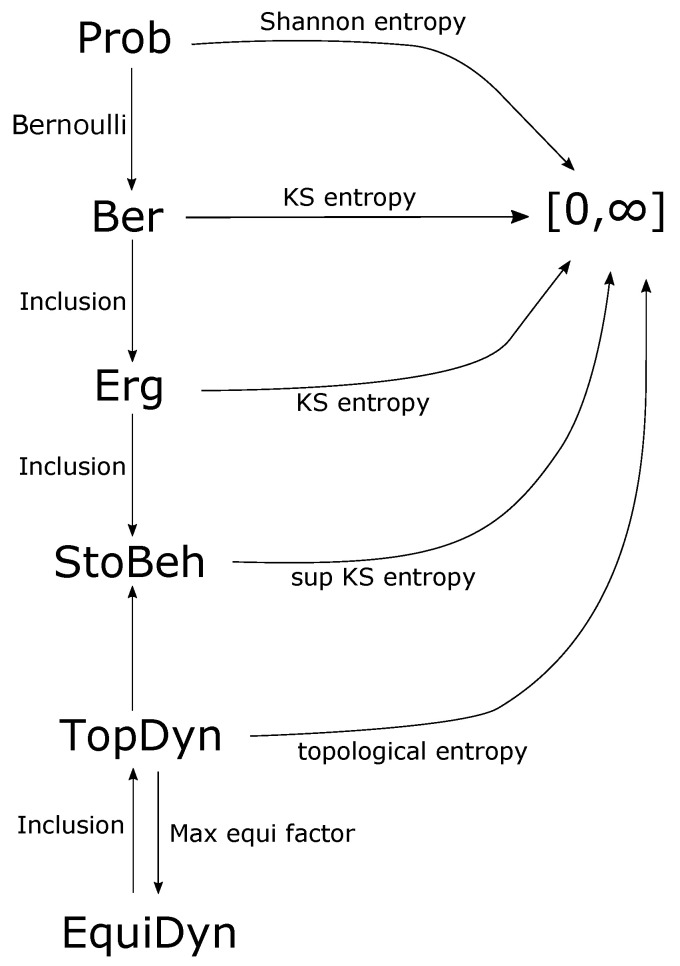
A summary of the categories and functors discussed in this article.

By a few examples we sketch how category theory can formulate in a natural way deep results of ergodic theory or topological dynamics, suggest the natural quantities or concepts to consider, and in some cases provide a simpler and more powerful proof. We also suggest that the ‘right’ category for measure-theoretic systems is **StoBeh**, the category of convex families of measures invariant under a given transformation of a measurable space. It is the right one for its intrinsic properties (existence of limits) as well as for its expressive power. Indeed it allows a natural framework for open systems theory (control theory), where we can formulate control problems and interconnection of behaviours in a unified way. See Figure 1 for a summary of the categories considered in this article and their relationships.

Much remains to be done to validate the ideas discussed in this paper. For example, a categorical proof of deep, classic or new, theorems in ergodic theory would be a landmark result. Also, formulating useful information-theoretic quantities that can capture the difficulty of control of networked open systems is in our view an interesting line of research.

## Appendix A. Characterization of KS and Shannon Entropies as Monoidal Functors

We see the category **Ber** of Bernoulli shifts and factor maps as monoidal, with the direct product as tensor product and the one-point Bernoulli shift as neutral object. We also see the nonnegative reals, augmented with +∞ as a monoidal category, with a single arrow a→b if and only if a≥b, the addition as tensor product, and 0 as neutral object. In this context, a monoidal functor from **Ber** to [0,+∞] is a functor that preserves the tensor product and the neutral object. We denote h(B) for the Kolmogorov–Sinai (KS) entropy of *B*, measured in bits. The elementary properties of KS entropy show that *h* is such a monoidal functor. The following theorem shows that it is essentially the only one.

**Theorem** **A1.**
*Consider an arbitrary monoidal functor F from ***Ber*** to [0,+∞]. Assume that F is non-trivial, in that it assigns a non-zero, non-infinite value to at least one finite-alphabet Bernoulli shift.*

*Then there is a real λ>0 such that F(B)=λh(B), for any object B of Ber. In short, F=λh.*

The proof goes as follows.

**Proof.** Consider $B_{0}$ the Bernoulli shift for which $0<F(B_{0})<+\infty$. Now consider any other Bernoulli shift *B*. We show that from the single knowledge of $F(B_{0})$, one can determine uniquely the value of F(B) in [0,+∞]. For any positive integers k,ℓ, either there is a factor map from the tensor power $B_{0}^{k}$ to $B^{\ell }$, or there is a factor map from $B^{\ell }$ to $B_{0}^{k}$, or possibly both. Since the functor transforms the factor maps into ≥ relations, in the first case we deduce that $F\left(B_{0}^{k}\right)\geq F\left(B^{\ell }\right)$, in other words $F\left(B\right)/F\left(B_{0}\right)\leq k/\ell$. In the second case we deduce $F\left(B\right)/F\left(B_{0}\right)\leq k/\ell$. Repeating the procedure for all k,ℓ, we determine a sequence of rational upper or lower bounds for $F\left(B\right)/F\left(B_{0}\right)$. Three cases occur:

- If $F\left(B\right)/F\left(B_{0}\right)$ is upper bounded by every positive rational, then F(B) must be zero;
- if $F\left(B\right)/F\left(B_{0}\right)$ is lower bounded by every positive rational, then F(B) must be +∞;
- if $F\left(B\right)/F\left(B_{0}\right)$ has a rational lower bound and upper bound, then one can produce increasingly refined upper and lower bounds, and assign a real positive value to F(B).

This defines *F* uniquely. On the other hand, the functor λh, multiple of the KS entropy functor *h* with coefficient $\lambda =F\left(B_{0}\right)/h\left(B_{0}\right)$, has the same value as *F* in $B_{0}$. Therefore F=λh, the proof is complete. As we have now characterized KS entropy as functor from **Ber** to [0,+∞], we now show how to extend it uniquely as a functor from all **Erg** to [0,+∞]. We first consider **BerFin**, the category of finite-alphabet Bernoulli, in order to have a small category. Since [0,+∞] is complete as a category (limits are suprema), we can create a right Kan extension of h:BerFin→[0,+∞] along the inclusion functor into **Erg**, see e.g., Theorem 3.7.2 of Borceux [4]. The general construction of the extended functor, in the present instance, turns out to map system *A* the supremum of h(B) for all finite-symbol Bernoulli factors *B* of *A*. By Sinai’s theorem, this extended functor is none but the KS entropy for general systems in **Erg**, as expected. □

## Appendix B. Maximal Equicontinuous Factor Systems from the General Adjoint Functor Theorem

We consider **EquiDyn**, the category of equicontinuous systems, full subcategory of **TopDyn**.

**Theorem** **A2.**
*The inclusion functor of ***EquiDyn*** into ***TopDyn*** has a left adjoint functor, which maps a dynamical system A to its maximal equicontinuous factor E(A) with the factor map A→E(A) as unit as the adjunction.*

We apply the General Adjoint Functor Theorem to build the adjunction, which we state as follows [4].

**Theorem A3** (General Adjoint Functor Theorem)**.**
*Let F:C→D be a functor between two categories C and D. Assume that C has all small limits. Then F has a left-adjoint functor if and only if it preserves all small limits and satisfies the Solution Set Condition. The Solution Set Condition states that for any object B in D there is a set of arrows $f_{i}:B\to F\left(A_{i}\right)$ such that any arrow g:B→F(A) can factored as $f=F\left(f\right)\circ f_{i}$ for some $f_{i}$ and some arrow $f:A_{i}\to A$ in C.*

To elucidate the intuition of the solution set condition in the general adjoint functor theorem, note that if the solution set was reduced to a single arrow $f_{0}:A\to A_{0}$ and the factorization of any g:B→F(A) through $f_{0}$ was unique, then the existence of a left adjoint *G* to *F* would be straightforward according to one of the classical characterizations of adjoint functors, with $G\left(B\right)=A_{0}$. The proof of the general adjoint functor theorem shows how to reduce the solution set to a single arrow.

We now prove Theorem A2.

**Proof.** First we check that **EquiDyn** has all (small) limits, which are the same in **EquiDyn** and in **TopDyn**. It clearly has all products, which are the usual Cartesian product with the product topology (see Chapter 2 of [10]). Equalizers are always monomorphisms, hence subsystems. But we know that equicontinuous systems are closed under subsystems. Thus we have all equalizers, hence all limits, trivially preserved by the inclusion. The solution set condition is verified as follows. For a dynamical system *A* in **TopDyn** we need to find a set of arrows from *A* to equicontinuous systems through which every arrow *f* from *A* to an equicontinuous system may factor. We observe that every such arrow *f* will factor through its image, thus we only need to look at surjective arrows, and consider equicontinuous factors. Clearly, the cardinality of a factor of *A* is at most the cardinality of *A*, therefore we can collect all equicontinuous factors of *A* into a set (rather than a proper class). Therefore the Solution Set Condition is met, and we can apply the general adjoint functor theorem. The fact that the adjoint functor creates the maximal equicontinuous factor is obvious by universality of the adjunction. The proof is complete. We note that the only ingredients specific to equicontinuity are invariance under subsystems and arbitrary products. The result can therefore be repeated to any subcategory of systems exhibiting these properties. □
